# A regime shift in the Sun-Climate connection with the end of the Medieval Climate Anomaly

**DOI:** 10.1038/s41598-017-11340-8

**Published:** 2017-09-11

**Authors:** D. A. Smirnov, S. F. M. Breitenbach, G. Feulner, F. A. Lechleitner, K. M. Prufer, J. U. L. Baldini, N. Marwan, J. Kurths

**Affiliations:** 1 Saratov Branch of V.A. Kotel’nikov Institute of Radio Engineering and Electronics of the Russian Academy of Sciences, 38 Zelyonaya St., Saratov, 410019 Russia; 2 Institute of Applied Physics of the Russian Academy of Sciences, 46 Ulyanova St., Nizhny Novgorod, 603950 Russia; 3 Department of Earth Sciences, University of Cambridge, Downing Street, Cambridge, CB2 3EQ UK; 4 Potsdam Institute for Climate Impact Research, P.O. Box 60 12 30, 14412 Potsdam, Germany; 5 Department of Earth Sciences, University of Oxford, Oxford, OX1 3AN UK; 6 Department of Anthropology, University of New Mexico, Albuquerque, NM 87106 USA; 7 Department of Earth Sciences, Durham University, Science Labs, Durham, DH1 3LE UK; 8 Present Address: Institute for Geology, Mineralogy, and Geophysics, Ruhr-University Bochum, Bochum, 44801 Germany

## Abstract

Understanding the influence of changes in solar activity on Earth’s climate and distinguishing it from other forcings, such as volcanic activity, remains a major challenge for palaeoclimatology. This problem is best approached by investigating how these variables influenced past climate conditions as recorded in high precision paleoclimate archives. In particular, determining if the climate system response to these forcings changes through time is critical. Here we use the Wiener-Granger causality approach along with well-established cross-correlation analysis to investigate the causal relationship between solar activity, volcanic forcing, and climate as reflected in well-established Intertropical Convergence Zone (ITCZ) rainfall proxy records from Yok Balum Cave, southern Belize. Our analysis reveals a consistent influence of volcanic activity on regional Central American climate over the last two millennia. However, the coupling between solar variability and local climate varied with time, with a regime shift around 1000–1300 CE after which the solar-climate coupling weakened considerably.

## Introduction

A very important problem in climate science is to understand and evaluate the relative contributions of natural factors to the observed global and regional climate variations on decadal to centennial time scales. Because instrumental records do not extend beyond the last three centuries, they cannot inform us on the low-frequency part of these climate variations and their links to external and internal climate forcings. Therefore, any link between climate change and types of forcings needs to be extracted from palaeoclimate archives, such as ice cores, tree rings, or stalagmites^1^. On decadal and multi-decadal time scales, changes in solar irradiance^2, 3^ and volcanic activity^4–6^ are the most potent drivers of natural variability in the natural climate system.

The influence of solar irradiance and volcanic activity on natural climate variability has been subject of intensive research (e.g. refs 7–10) including directional coupling estimation from instrumental records over the last 150 years (see Supplementary Section 1 and references therein). Until now however, specific methods for inferring directional couplings from observational data were not systematically used to analyze the causal interrelationships between climate and solar or volcanic activity on longer (centennial to millennial) time scales. The latter is necessary, for example, to quantify the variable effects and individual contributions of solar and volcanic forcings through time, to better understand the forcing mechanisms and to improve predictive climate models. There has been considerable interest in how extended periods of low solar activity are linked to climatic changes^11, 12^ and what this implies for the future^13, 14^. A notable example is the 17^th^-century grand solar Maunder Minimum^15^ and its link to a period of intense cold within the Little Ice Age in the Northern Hemisphere^16^. Recent research suggests that the particular cold spell around the time of the Maunder Minimum was triggered by a series of volcanic eruptions and sustained by atmospheric and oceanic feedbacks rather than changes in solar irradiance^17, 18^. Indeed, evidence from a network of tree-ring chronologies from the North American taiga suggests a drastic regime shift to lower summer temperatures following a series of tropical eruptions in the late 13^th^ century^19^.

Furthermore, systematic investigations of causal relations between natural forcings and global and regional climate can shed light on potential variations of these links over time and space^20^. It is often implicitly assumed that causal couplings remained constant through time. A rigorous analysis of the time-dependence of these couplings however is vital for a deeper understanding of the complex influence of solar and volcanic activity on global and regional climate, especially if lessons for present-day conditions are inferred from past climatic changes.

**Figure 1:**
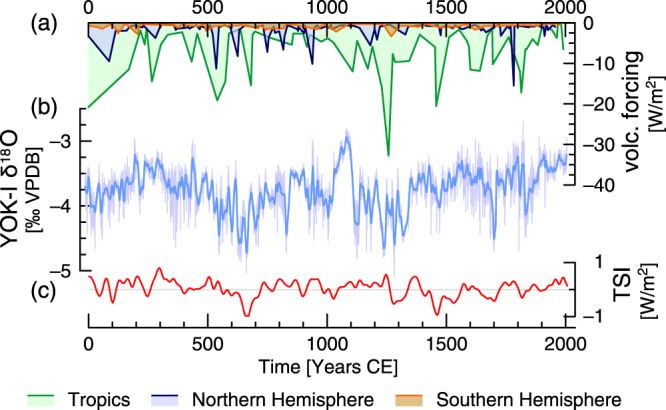
Time series of (**a**) volcanic forcing originating in the tropics, and northern and southern hemispheres^8^, (**b**) YOK-I δ^18^O (original and Gaussian kernel smoothed data – grey and blue lines, respectively)^6, 21, 30^, and (**c**) solar forcing (red line)^22^.

In this study, we investigate the temporal evolution of the regional influence of solar and volcanic activity on paleorainfall in southern Belize using an extended, temporally resolved Wiener-Granger causality analysis of a 2000 year long rainfall proxy record derived from Yok Balum Cave in southern Belize, Central America. The stable isotope record from stalagmite YOK-I^6, 21^ represents one of the most highly resolved and best dated datasets available from Central America for the last two millennia (Fig. 1b). The YOK-I stable oxygen (δ^18^O) and carbon (δ^13^C) isotope ratio time series give detailed insights in local rainfall and infiltration changes in southern Belize with near-annual temporal resolution. This proxy record is well suited for the study of dynamic link changes between forcing and regional terrestrial climatic response, due to its exceptional chronological control, high proxy resolution and location near the northern limit of modern summer Intertropical Convergence Zone (ITCZ) extent. In southern Belize, rainfall is driven by the annual migration of the ITCZ, which reaches its northernmost extent during the boreal summer months. Stronger summer insolation results in a warmer Northern Hemisphere and higher Belizean rainfall totals due to northward displacement of the ITCZ. Enhanced volcanic activity (and consequently higher atmospheric aerosol content) in the Northern Hemisphere, on the other hand, leads to atmospheric cooling and a southward shift of the ITCZ, resulting in lower rainfall over southern Belize^6^. By analyzing the YOK-I rainfall proxy data we are able to obtain quantitative empirical estimates of the strength of these relationships and to reveal their temporal variation.

We focus on the causal links between solar activity, volcanic forcing, and climatic changes documented in the YOK-I record, and on possible variability of the strength of these links through time. For this analysis, solar activity changes over the last 2000 years are inferred from a reconstruction of total solar irradiance (TSI) from ^10^Be concentrations in Greenland ice cores^22^ (Fig. 1c). To assess the influence of volcanic aerosols the most recent ice core-based estimate of natural (volcanic) atmospheric aerosol loadings (sulfate accumulation) is used^8^ (Fig. 1a). To unravel the hidden link dynamics, we employ the specialized Wiener-Granger causality analysis and assess the associated uncertainties using both Fisher’s *F*-criterion and a Monte Carlo-based significance test (see Section “Data and Methods” below). We compare the results with a more widely known and less specific approach to coupling analysis based on the cross-correlation function (CCF).

As a measure of the influence of an observed process *y* (solar or volcanic activity) on another observed process *x* (local climate proxy), we use the Wiener-Granger causality characteristic ${G}_{y\to x}$ which assesses how strongly the future of *x* depends on the past of *y* given the past of *x* ^23^. This measure is defined as prediction improvement of the autoregressive models fitted to the data from *x* only or from both *x* and *y*. To account for possible dating errors, we calculate ${G}_{y\to x}(s)$ where *s* stays for a trial time shift of the signal *x* to the past by *s* years, and ${G}_{y\to x,{\rm{\max }}}$ is the value maximal over an appropriate range of *s*. The results are compared to Pearson cross correlation function (CCF) ${K}_{yx}(l)$ where positive lags *l* indicate that *y* leads *x*, and the absolute CCF value maximized over an appropriate range of *l* is $|{K}_{yx}{|}_{\max }$. Further details including assessment of the statistical significance levels (*p*-levels, both pointwise and corrected for multiple testing) are given in “Data and Methods” below.

## Results

**Figure 2:**
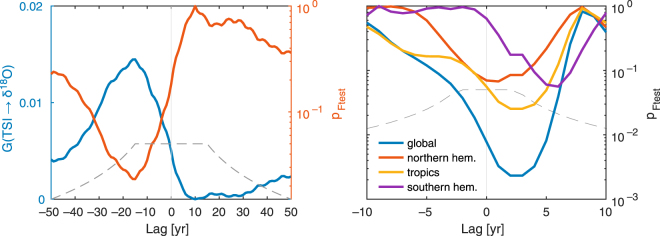
Coupling between Central American regional climate (represented by YOK-I δ^18^O) and solar forcing (left) and volcanic forcing (right) as estimated by a Granger causality analysis. Asymptotic point-wise significance level (left, red line) of the Granger causality estimates (left, blue line) indicate a significant influence of the solar forcing on the regional climate with a lag of −15 yr due to dating errors (Bonferroni-corrected significance level of *p* = 0.05 is marked by grey dashed line). The point-wise significance level for the volcanic forcing (right) indicates influences from the global volcanic activities and topical eruptions, but no significant influence from volcanoes of the Northern or Southern Hemisphere (grey dashed line: Bonferroni-corrected significance level of *p* = 0.05).

The Wiener-Granger causality test confirms the causal influence of solar irradiance on regional moisture availability in southern Belize as reflected in the YOK-I δ^18^O time series (Fig. 2). The maximal link strength is found at a time lag of YOK-I δ^18^O of –15 years. This lag corresponds to an (illogical) situation of proxy leading the forcing, like that found in an earlier study comparing solar activity and speleothem based paleoclimate variation^24^. This lag is in the range of dating uncertainties in the time series involved and most likely results from uncertainties in the TSI age model. The lag is therefore considered only as resulting from these uncertainties, and we use the logical interpretation of the forcing towards the proxy (and not *vice versa*) without over-interpreting the exact value of the lag. The coupling is significant at (asymptotic) Bonferroni-corrected *p* < 0.05. Using the ice core-based volcanic sulfate data, the Wiener-Granger analysis also confirms a robust influence of volcanic activity on regional moisture availability in southern Belize, with a maximum volcanic influence at a time lag of 1 to 2 years and a significance level lower than *p* = 0.003 according to the *F*-test. Such 1 to 4 years lagged regional response to volcanic forcing is commonly found in paleoclimate records, e.g. ref. 19. The dating uncertainties in the volcanic forcing data are lower than in the solar proxy record. In order to identify the latitudinal range with the strongest impact of volcanic forcing on southern Belizean rainfall, we separated the test into volcanic activity in the tropics, Northern, and Southern Hemisphere, using the volcanic forcing reconstruction of *Sigl et al*.^8^. Our analysis corroborates earlier findings^25^ that tropical eruptions have the strongest impact on Central American climate (*p* < 0.05), whereas eruptions originating in the Northern and Southern Hemisphere have only slight influences (vanishing significance, *p* > 0.05). Interestingly, the regional hydroclimate seems to respond to tropical volcanic forcing somewhat later compared to Northern Hemisphere forcing. We note that an earlier study on volcanic forcing of rainfall at Yok Balum Cave^6^ categorized eruptions slightly differently, by not considering tropical eruptions as a separate category but instead attributed them to either the Northern Hemisphere or Southern Hemisphere. This current study complements Ridley *et al*.^6^ by also assessing how the latitude of the eruption affected the climate response, but does not distinguish the hemisphere of low latitude eruptions.

**Figure 3:**
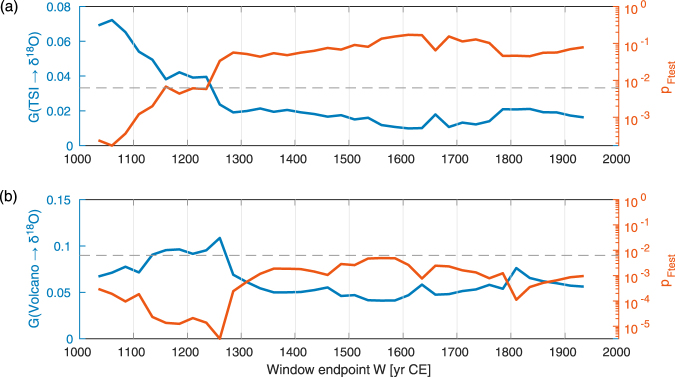
Temporal variation of the forcing on the regional climate represented by δ^18^O. (**a**) Wiener-Granger causality for TSI forcing YOK-I (blue line), and (**b**) Wiener-Granger causality for volcanic aerosols forcing YOK-I (blue line) versus the moving window endpoint *W*. The red curves in (**a**) and (**b**) represent the *p*-values as derived from the *F*-test, revealing a more or less constant highly significant volcanic forcing of the climate, but solar forcing decreasing after around 1300 CE.

**Figure 4:**
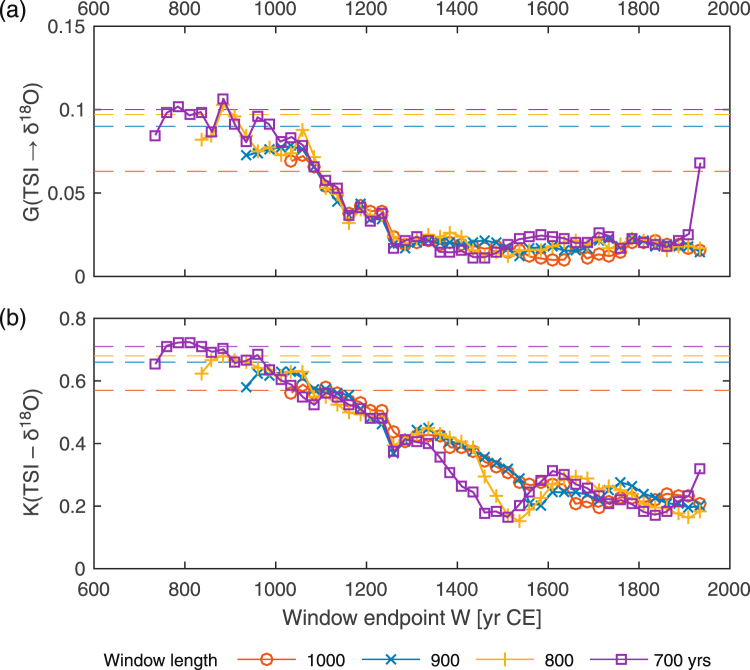
The Wiener-Granger causality estimates for the TSI and δ^18^O variations (**a**) and maximal CCF between these processes (**b**) in moving windows of different lengths. The dashed lines show the corresponding Monte Carlo-based 0.95-percentiles for the quantities maximized over all subsequent time windows within a 2020 yr interval for the uncoupled model (1).

To extract information on the variation of the causal coupling over time, we calculated the coupling characteristics within 1000-year moving windows and use the Wiener-Granger statistic ${G}_{y\to x,{\rm{\max }}}$. The window length represents a compromise between statistical robustness and temporal resolution of the Wiener-Granger technique and the maximum lag is considered sufficient to cover both physical delays between forcing and climate as well as dating uncertainties. The analysis applied on the solar data reveals a significant change of the influence of solar irradiance occurring between 1000 and 1300 CE (Fig. 3a). Before this period (50 BCE to 1050 CE), the Wiener-Granger causality test shows a strong influence of solar irradiance on regional moisture (pointwise significant at *p* < 0.001 according to *F*-test). After 1300 yr CE, this influence diminished to being barely significant (pointwise *p* ≈ 0.1). Plotting the values of ${G}_{y\to x,{\rm{\max }}}$ for moving windows of different lengths *L* versus the window endpoint *W* (Fig. 4a, solid lines) helps to justify the localization of the transition interval. The dashed lines in Fig. 4a represent the Monte-Carlo based 0.95-percentile for the quantity ${G}_{y\to x,{\rm{\max }}}$ maximized over all windows within the entire 2020-years interval, and we can again reject the hypothesis of *no coupling* at *p* < 0.05 since ${G}_{y\to x,{\rm{\max }}}$ exceeds that percentile for any window length in the range from 700 to 1000 yrs. The largest, significant values of ${G}_{y\to x,{\rm{\max }}}$ are observed for several early time windows (in particular, 100 CE to 1100 CE). The transition interval between stronger and weaker couplings in terms of *W* appears *the same* (1000 CE to 1300 CE) *for any moving window length in the above range*, i.e. ${G}_{y\to x,{\rm{\max }}}$ starts to decrease only when the data later than 1000 CE start to fall into the time window used for the estimation. This is evidence for a change of the coupling strength taking place in the latest part of the time interval (0–1300 CE), i.e. within the interval of [1000 CE – 1300 CE]. If the coupling would have changed in earlier parts, e.g. (0 CE – 300 CE), the decrease of ${G}_{y\to x,{\rm{\max }}}$ for different window lengths *L* would have been apparent at different *W* (at smaller *W* for smaller *L*).

The fact that significant coupling estimates are obtained for earlier time windows (roughly, in the first millennium CE) and insignificant for later windows (in the second millennium CE) is a sign of change in coupling strength, but not direct evidence for the statistical significance of such change. The latter might be a result of statistical fluctuations due to short window lengths, i.e. insignificant coupling estimate for the second millennium CE might be due to noise effect rather than due to weaker coupling. To address this question directly, we performed a Monte Carlo test against the model (1) with constant parameters by calculating the range ${\rm{\Delta }}{G}_{y\to x,{\rm{\max }}}$ over all *W* at fixed *L* (see “Data and Methods”). It allowed rejection of the hypothesis of constant parameters and, hence, to infer a statistically significant decrease in coupling strength at the significance level of *p* = 0.1. This result provides statistically robust (“very probable” using the IPCC nomenclature^26^) support for the hypothesized regime shift over the time interval of interest.

**Figure 5:**
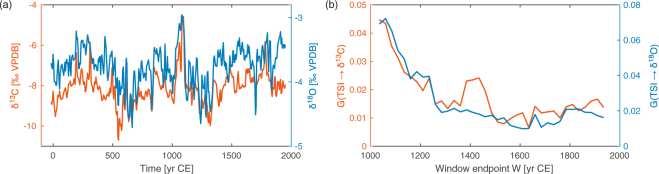
Comparison of the results for the two isotope records from the YOK-I stalagmite: (**a**) time series of δ^13^C (red line, left axis) and δ^18^O (blue line, right axis), (**b**) moving window based Wiener-Granger causality estimates between TSI and the two isotope records.

Similar results are found for the YOK-I δ^13^C time series (Fig. 5a); although not as clearly as for δ^18^O (Fig. 5b), these results confirm previous studies suggesting that solar forcing and YOK-I δ^13^C become decoupled around 1250 CE^27^. The discrepancy between δ^13^C and δ^18^O may result from multiple controls on δ^18^O, including moisture source, moisture amount, and height of the convective column^28^.

To test the robustness of the results, we performed the same analysis using another solar reconstruction based on the Δ^14^C data compiled from various sources (IntCal13, Supplementary Figure S7)^29^. Overall, the same qualitative conclusion about solar influence decreasing during the interval 1000 to 1300 CE can be drawn (Supplementary Section S5). Moreover, the maximal WG causality for early windows is achieved at physically meaningful positive lag of 10 yr (Supplementary Figure S9) in contrast to the negative lag for the above TSI reconstruction (Fig. 2) supporting the hypothesis of the possible dating error in the TSI record. Still, statistical significance of the results for the Δ^14^C data is worse than for TSI, which is partly explained by the presence of strong slow components (time scales of several hundreds of years) in the Δ^14^C data. Removal of such trends would not be straightforward and may introduce statistical artefacts. Comparison with other solar reconstructions and other speleothem data from Central America is not currently possible since other available proxy records do not satisfy the conditions of sufficiently long duration (at least 2000 years), high temporal resolution (sampling interval not greater than 5 yrs) and stationarity (no strong slow components with time scales of 500 yrs or longer). To our knowledge, these requirements are currently met only by the used YOK-I and TSI data.

The windowed analysis does not reject the hypothesis that the influence of volcanic activity on climate remains invariant through time (Fig. 3b). This conclusion is based on the Monte Carlo tests with the model (2) and window length *L* = 1000 yrs. Although the hypothesis of *no coupling* is confidently rejected at the level of *p* < 0.001, the hypothesis of constant parameters is not rejected at any reasonably small significance level suggesting that the volcanic influence on climate remains more constant in time than the detected time-changing solar influence. For a vivid comparison, note that the value of ${G}_{y\to x,{\rm{\max }}}$ changes between the first millennium CE and the second millennium CE by the factor of four for the solar activity (Fig. 3a) and only twice for the volcanic activity (Fig. 3b). This indicates a quite robust response of regional atmospheric circulation and the regional hydroclimate to volcanic aerosol forcing, supporting previous interpretations^6^.

These results regarding the presence and decrease of coupling between solar activity and climate are confirmed by common CCF analysis. Namely, the CCF reveals an overall general correlation at a Bonferroni-corrected significance level of *p* = 0.04 and indicates a decreasing correlation between the TSI and YOK-I δ^18^O (Fig. 4b) as well as δ^13^C with time. For example, the window length of 700 years shows that starting around 800 CE the maximal correlation $|{K}_{yx}{|}_{\max }$ gradually decreases from 0.7 to values below 0.3 at around 1400 CE (Fig. 4b). However, the change is more gradual than that of the Wiener-Granger causality reflecting the lower sensitivity of CCF analysis to changes in coupling strength. Thus, the CCF analysis identifies the time interval of change less clearly, explainable by the fact that CCF does not specifically detect coupling direction and consequently the CCF value as a characteristic of the causal coupling strength is ambiguous. Over the same period, the correlation between global volcanic activity and δ^18^O remains relatively constant (Supplementary Figure S12d). Further details are given in Supplementary Sections S3.1, S3.2, S4.1 and S6.

## Discussion and Conclusions

The intriguing weakening of the influence of solar irradiance on rainfall over southern Belize following the advent of the Little Ice Age strongly suggests that the impact of solar insolation on the regional hydroclimatic dynamics in tropical Belize was not constant during the last two millennia. Increased solar insolation enhances Northern Hemisphere warming, resulting in northward ITCZ displacement and consequently more rainfall in the study region. Enhanced atmospheric aerosol concentrations (volcanic or anthropogenic) cool the NH sufficiently to force the ITCZ southward^6^. ITCZ position reflects a delicate balance between the opposing effects of solar and (mainly) volcanic forcing. The geographical location of the Yok Balum Cave at the northern realm of the ITCZ makes proxy records from this cave sensitive to changes in the meridional position of the ITCZ. Our study reveals a rather constant influence of volcanic forcing, but a changing influence of the solar forcing (according to our rigorous statistical analysis over 800–1000 yrs intervals). Large volcanic eruptions are not uncommon, and the clustering in time of large eruptions over the last 2000 years is largely down to chance. These volcanic effects are superimposed on atmospheric conditions that are otherwise modulated by solar forcing. Before 1200 CE, solar forcing significantly contributed to the regional climate variability, resulting in enhanced rainfall over the Caribbean region, including southern Belize. With the onset of the Little Ice Age around 1200 CE, the solar influence decreased, resulting in drier regional climate conditions. The influence of volcanic aerosols stems mainly from tropical eruptions, with less severe impact of volcanic activity in both the Northern and Southern Hemispheres. Our results emphasize the importance of potential changes of the strength of the solar impact, relative to other forcings, on climate when interpreting paleoclimate variability.

In this study we introduce Wiener-Granger causality adjusted for possible dating errors as a method for inferring directional coupling, and develop an appropriate significance test for use on palaeoclimate datasets. This approach reveals a changing solar influence on rainfall in southern Belize, with the solar influence considerably decreasing after approximately 1300 CE. In contrast, the impact of volcanic activity on the regional rainfall remains consistent over the last two millennia, thus increasing its importance relative to solar forcing after 1300 CE. The technique is promising, and future studies should use similar approaches to further deconstruct seemingly complex couplings involving multiple possible forcings.

## Data and Methods

### Data

In our analysis, we use previously published δ^18^O and δ^13^C data from stalagmite YOK-I from Yok Balum Cave, southern Belize^6, 21, 30^. Stalagmite δ^18^O at this cave site has previously been interpreted as reflecting rainfall amount and the δ^18^O of precipitation^21, 28^. Hydroclimate in southern Belize is driven by the seasonal migration of the ITCZ, resulting in distinct rainfall seasonality (wet summers, dry winters). Moisture at Yok Balum Cave is predominantly derived from the adjacent Gulf of Honduras and the Caribbean Sea, with increasingly important contributions from more distal moisture sources (i.e., tropical Atlantic) during the boreal summer months^21^. The influence of large storm cells and high intensity rain during the summer rainy season results in more depleted δ^18^O values registered in surface waters and in a clear “amount effect”^21, 31^. Changes in moisture source have some influence on rainfall δ^18^O over the course of the year, with enhanced Rayleigh distillation of moisture in more distally sourced storms during the summer months, contributing to the summer depletion in δ^18^O. However, the amount effect appears to be the dominant driver of precipitation variability, and stalagmite δ^18^O can thus be interpreted as a proxy for rainfall amount^21, 31^.

Stalagmite δ^13^C largely reflects the effective rainfall amount at the site, and hydrological conditions in the karst system^6, 27^. Dry conditions in southern Belize result in less negative δ^13^C values, due to reduced soil bioproductivity, increased prior calcite precipitation and cave ventilation, and a higher proportion of bedrock carbon to the dripwater. Conversely, more negative δ^13^C values reflect wetter conditions at the site. By validating the analysis performed on YOK-I δ^18^O with δ^13^C, we can confirm that the proxy response to solar/volcanic forcing is indeed reflecting a reduction in precipitation at the study site, and not simply a change in moisture source isotopic composition upstream of the cave (as suggested for caves in China, e.g. refs 32–34).

### Data availability

YOK-I data^30^: http://doi.org/10.5880/pik.2017.004. TSI data^22^: ftp://ftp.ncdc.noaa.gov/pub/data/paleo/climate_forcing/solar_variability/steinhilber2009tsi.txt. Volcanic forcing data^8^: http://www.nature.com/nature/journal/v523/n7562/abs/nature14565.html#supplementary-information.

### Methods

The concept of Wiener-Granger causality was originally developed for mathematics^35^ and econometrics^23^, but is becoming widely used for characterizing directional coupling in physical and other research disciplines^36–38^. Influence of a process *y* on a process *x* is usually quantified by comparing prediction errors of autoregressive (AR) predictive models fitted to the data from *x* only or from both *x* and *y* (see Eqs (S1) and (S2) in Supplementary Section S3.3). Below we construct one-step-ahead AR models where the time “step” is the sampling interval Δ*t*, which is equal to 5 years in our study. To estimate the Wiener-Granger causality in the direction *y → x*, we find the least possible mean-squared prediction error ${\sigma }_{x}^{2}$ of the individual AR model for *x* (where the next *x* value is predicted based on the history of *x*) and the error ${\sigma }_{x,y}^{2}$ of a joint AR model, where the next *x* value is predicted based on the histories of both *x* and *y*. The normalized prediction improvement ${G}_{y\to x}=({\sigma }_{x}^{2}-{\sigma }_{x,y}^{2})/{\sigma }_{x}^{2}$ is the basic measure of coupling “strength”. If ${G}_{y\to x}$ is significantly greater than zero, the influence *y* → *x* can be inferred. Statistical significance can be estimated via an (asymptotic) *F*-test (see Supplementary Section S3.3) if the time series is sufficiently long. This model-based approach often enhances the sensitivity of the coupling analysis as compared to the CCF or traditional regression analysis, since the internal dynamics of the studied processes are considered in ${G}_{y\to x}$.

To incorporate the possible (mean) dating error, we shift the signal *x* by *s* years (positive *s* means shifting *x* to the past) and calculate ${G}_{y\to x}(s)$ searching for its maximum. Without dating errors, only positive lags of the maximum are expected, corresponding to an influence of solar or volcanic activity on the regional climate (represented by the paleoclimate proxy *x*). However, considering relative dating uncertainties complicates the situation by moving the maximum in any direction from its physically justified location at $s\ge 0$. Errors associated with even the earliest dates within the YOK-I record are less than 20 years, compared with basic time scales of the considered processes of no more than 25 to 30 years (Supplementary Section S2) that lead to the respective statistical fluctuations of the maximum point. Combined, these two factors could shift the maximum of ${G}_{y\to x}(s)$ by no more than 50 yrs. Maximization over the appropriate range of *s* provides ${G}_{y\to x\max }=\mathop{\max }\limits_{-50yr\le s\le 50yr}{G}_{y\to x}(s)$ and reduces the effects of dating errors as large as those expected for the data under study. More details on this adjustment of the method are given in^39^.

We compare the Wiener-Granger causality estimation results with the standard approach for correlation analysis by calculating the Pearson cross correlation coefficient ${K}_{yx}(l)$ between the local climate proxy (*x*) and solar or volcanic activity (*y*) for different lags *l*. Positive values for *l* indicate that *y* leads *x*. Statistical significance of *K*_*yx*_ different from zero at given *l* can be estimated via an (asymptotic) *Z*-test assuming zero-mean Gaussian distribution of the CCF estimator (see e.g. ref. 40). Incorporating possible dating errors described above, we use mainly the maximal CCF value $|{K}_{yx}{|}_{\max }=\mathop{\max }\limits_{-50yr\le l\le 50yr}|{K}_{yx}(l)|$.

### Models and procedure for Monte Carlo significance testing

Along with the asymptotic *F*-test and *Z*-test including the Bonferroni correction for multiple testing^41^, we use Monte Carlo tests based on specific models in the form of first-order stochastic differential equations and random impulse processes, which reproduce basic statistical properties of the observed data. With these models, we get typical estimator values for the same time series length, sampling frequencies, and dating errors as those in the data under study, without the requirement of infinitely (or at least very) long time series as would be necessary for the asymptotic *F*-test. Moreover, these models allow simulations of different coupling conditions and testing against the hypothesis of *constant coupling* in addition to the *no coupling* scenario. Using moving windows of the length ranging from 500 to 1200 yrs, we test for potential changes in the coupling through time.

Such a bivariate model for the joint δ^18^O and solar activity variations reads

1 $$\begin{array}{c}dX=\frac{1}{{\tau }_{x}}Xdt+cYdt+d\xi (t),\\ dY=\frac{1}{{\tau }_{y}}Ydt+d\eta (t),\end{array}$$

where *Y* represents the solar activity underlying the observed signal *y*_*n*_ (TSI data), *X* represents the climate process underlying the observed signal *x*_*n*_ (δ^18^O variations), *τ*_*x*_, *τ*_*y*_ are relaxation times, *c* is the coupling coefficient, $d\xi (t)$ and $d\eta (t)$ are mutually independent Wiener processes with variances $2{D}_{\xi }dt$ and $2{D}_{\eta }dt$, respectively (${D}_{\xi }$ and ${D}_{\eta }$ are diffusion coefficients which determine the noise intensities). Replacing *dt* with the finite time interval Δ*t* = 1 month, setting *τ*_*x*_ = 20 yrs, *τ*_*y*_ = 25 yrs, $2{D}_{\xi }{\rm{\Delta }}t=2{D}_{\eta }{\rm{\Delta }}t=0.0004$, introducing irregular time sampling of *X* in accordance with the observed sampling frequency (see Supplementary Figure S1c), and performing the Gaussian kernel averaging of *X* with *σ* = 2.5 years and of *Y* with *σ* = 5 years (see Supplementary Section S2), we get the behavior of the model (1) quite close to the observed properties of the data. Since the decorrelation times of the processes are artificially increased by about 5 yrs due to the kernel averaging, we try the relaxation times for the underlying process in the range 20–25 yrs in the model (1). Setting the coupling coefficient *c*Δ*t* = 0, we simulate the case of uncoupled processes. Setting *c*Δ*t*=*const* > 0, we simulate a nonzero constant coupling.

For each set of the parameter values, dating errors, time series length, and settings of the sampling procedure, we generate an ensemble of 1000 time series of the length of 2020 yrs with a time step of 5 yrs and perform CCF and Wiener-Granger causality estimation exactly as for the paleoclimate data above. Then, we compare the estimates obtained for the paleoclimate data with percentiles of the distribution estimated from the respective 1000-member ensemble. Thereby, we reject or do not reject the hypothesis of no coupling or of constant coupling under which the model time series are simulated. Such an approach avoids any assumptions of a very long time series. Therefore, it is a useful complementary tool to assess reliability of the conclusions.

In the Monte Carlo tests for significance of volcanic activity influence, we use a model of the type (1) where the *y* variable mimics the properties of the observed volcanic data summarized in Supplementary Figure S3c,d. Namely, we take *y* to be the sum of Gaussian impulses and finally get the model

2 $$\begin{array}{c}dX=\frac{1}{{\tau }_{x}}Xdt+cYdt+d\xi (t),\\ Y=-\sum _{k=1}^{K}{A}_{k}\exp (-\frac{{(t-{t}_{k})}^{2}}{2{\sigma }^{2}}),\end{array}$$

where *t*_*k*_ are random instants of pulses with inter-pulse distance taken at random from the fit to the distribution in Supplementary Figure S3d, amplitudes of pulses *A*_*k*_ are taken at random from the fit to the distribution in Supplementary Figure S3c, and the kernel width is *σ* = 2.5 years.

## Electronic supplementary material

Supplementary Information
