# Supplementary material for: A regime shift in the Sun-Climate connection with the end of the Medieval Climate Anomaly
Source: Sci Rep. 2017 Sep 11;7:11131. doi: 10.1038/s41598-017-11340-8 (PMC5594009; doi:10.1038/s41598-017-11340-8)
Supplement: Supplementary file 1 — Supplementary Information [file 41598_2017_11340_MOESM1_ESM.pdf]

Supplementary Information for "A regime shift in the Sun-Climate connection with the end of the Medieval Climate Anomaly"

D. A. Smirnov[1,2], S. F. M. Breitenbach[3], G. Feulner[4], F. Lechleitner[5], K.M. Prufer[6], J. U. L. Baldini[7], N. Marwan[4], J. Kurths[4,2]

[1]Saratov Branch of V.A. Kotel'nikov Institute of Radio Engineering and Electronics of the Russian Academy of Sciences, 38 Zelyonaya St., Saratov 410019, Russia.

[2]Institute of Applied Physics of the Russian Academy of Sciences, 46 Ulyanova St., Nizhny Novgorod 603950, Russia.

[3]Department of Earth Sciences, University of Cambridge, Downing Street, Cambridge CB2 3EQ, UK, now Institute for Geology, Mineralogy, and Geophysics, Ruhr-University Bochum 44801 Germany

[4]Potsdam Institute for Climate Impact Research, P.O. Box 60 12 30, 14412 Potsdam, Germany.

[5]Department of Earth Sciences, University of Oxford, Oxford OX1 3AN, UK.

[6]Department of Anthropology, University of New Mexico, Albuquerque, NM 87106, USA.

[7]Department of Earth Sciences, Durham University, Science Labs, Durham DH1 3LE, UK.

Corresponding author: Dmitry A. Smirnov (smirnovda@yandex.ru)

Contents

Section S1: References to previous analyses of instrumental records.

Section S2: Details of the data and methods used for data preprocessing.

Section S3: Details of the cross-correlation and Wiener-Granger causality estimation between TSI and YOK-I $\delta^{18}O$ data with asymptotic tests for significance.

Section S4: Details of the same estimation as in Section S3, but with Monte Carlo tests for significance.

Section S5: Results for solar activity proxy based on the compiled archive (Intcal13).

Section S6: Details of the coupling estimation for volcanic activity and YOK-I $\delta^{18}O$ data.

S1. Note on the previous studies of instrumental records

In our manuscript we present a systematic statistical study of causal couplings between solar irradiance, volcanic activity, and natural climate variability over the last 2000 years, based on high-resolution paleoclimate records. Previously, similar questions were addressed with help of instrumental (meteorological) records covering the last century and a half. In particular, the influence of solar irradiance and volcanic activity variations on global surface temperature (GST) variations has been studied intensively, e.g. [S1-S13]. The sampling interval of the data (time step) used in those works was equal to 1 yr or less. We note that different authors commonly found relatively weak (on the border of statistical significance) influence of solar activity variations and somewhat stronger (more significant) influence of volcanic activity on GST, e.g. [S1, S2, S7-S11]. Despite the different data sources and sampling intervals, those results are in general agreement with our present findings of stronger volcanic relative to solar influence on climate variability in the second millennium AD obtained on the basis of paleoclimate records.

S2. Details of the data under study

As a characteristic of hydroclimate variations in Central America (tropical Atlantic), we use the speleothem $\delta^{18}O$ proxy signal from stalagmite YOK-I, southern Belize (Fig.S1,a) which reflects regional moisture history and precipitation processes [S14,S15]. As a proxy for solar activity, we consider total solar irradiance (TSI, Fig.S2,a) variations reconstructed from variations of ^{10}Be concentration in Greenland ice cores [S16]. As a proxy for volcanic activity, we use the indices from Ref. [S17] (Fig.S3,a) which represent radiative forcing resulting from global atmospheric loading from volcanic activity of Northern Hemisphere, Southern Hemisphere, and Tropics.

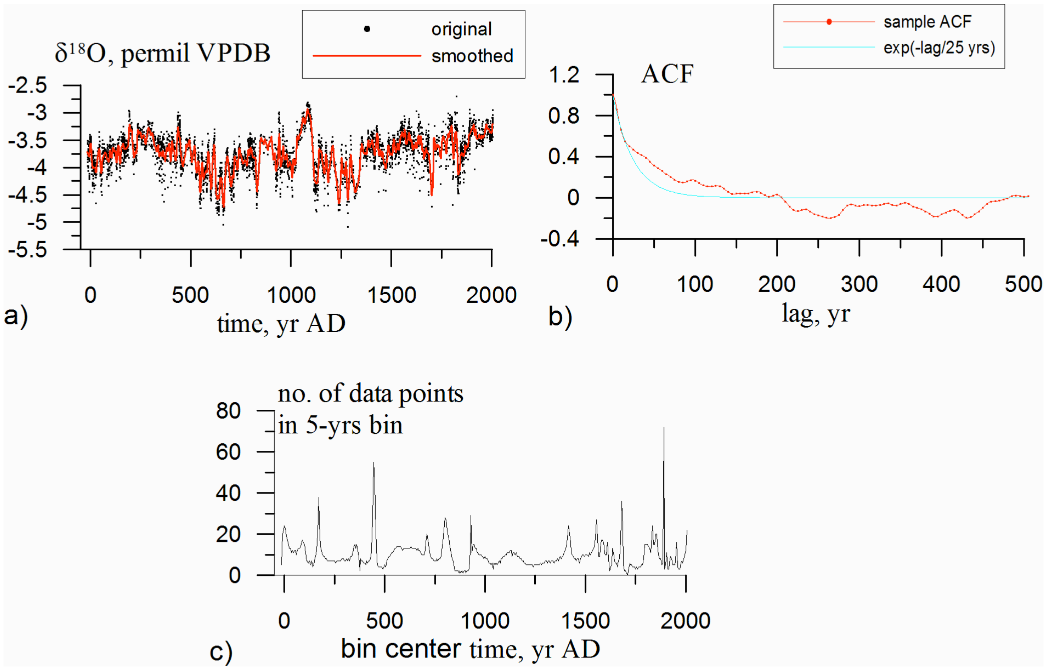

Figure S1. The data for $\delta^{18}O$ variations [S14,S15,S18]: a) the original time series [S18] (black) and smoothed by Gaussian kernel filter with σ = 2.5 years [S18] (red); b) the sample autocorrelation function (ACF) for the smoothed signal (red) with an imposed exponential function fitted over small lags; c) temporal variations of the non-uniform sampling frequency.

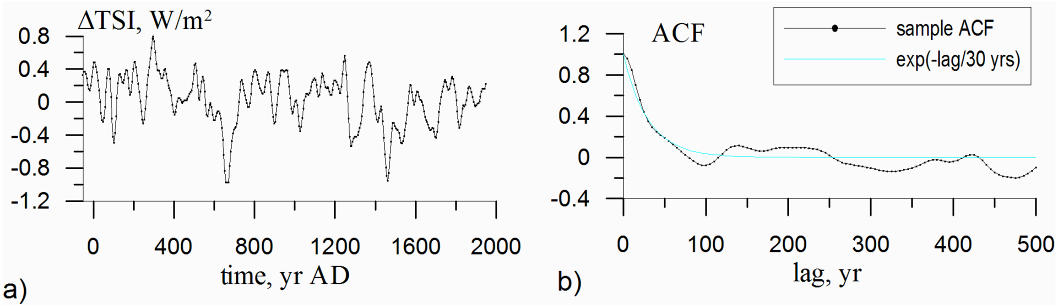

Figure S2. TSI variations [S16]: (a) an original 5-yr sampled signal; (b) its sample ACF (black) and an exponential fit over small time lags.

Denote the original data for the process of moisture variations as $\tilde{x}_n = \tilde{x}(\tilde{t}_n)$ where observation instants $\tilde{t}_n$ are distributed irregularly in time over the period 15 BC to 2005 AD. The sampling frequency ranges from one observation per month to one observation per 5 yrs (Fig.S1,c). Such changes are determined by the stalagmite growth rate depending on water supply and chemistry of the dripwater. Geochemical sampling is performed at regular distance intervals (e.g. 100 micron per sample), which leads to irregular sampling in time [S14]. The dating here is linked to a U/Th chronology with a mean error up to 13 years for ages about 2000 yrs BP [S14,S15].

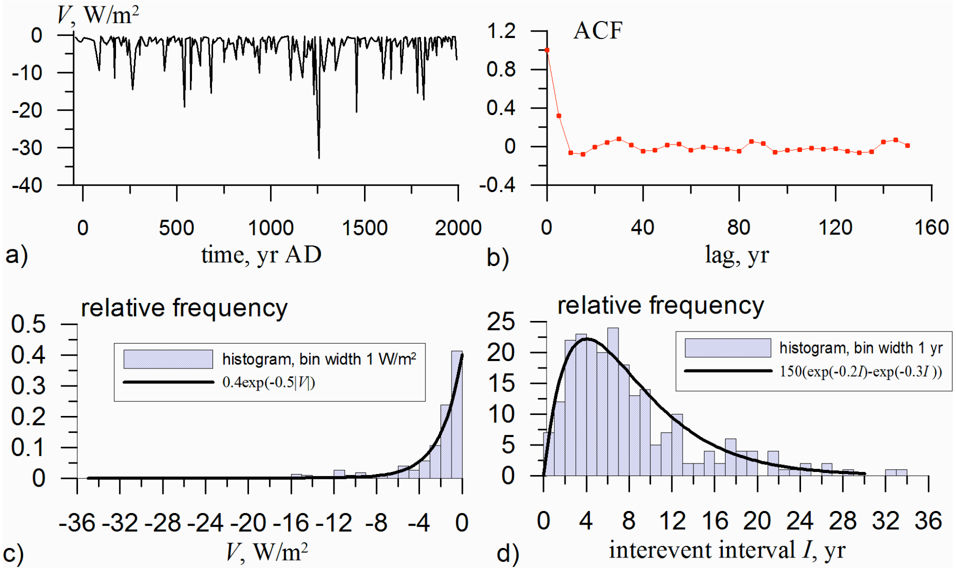

Figure S3. Volcanic activity data: a) global atmospheric loading V from all volcanoes, including NH, SH, and tropics [S17]; b) sample ACF for the signal smoothed by Gaussian kernel with σ = 2.5 yrs; c) amplitude distribution for the original data (gray) and the fit 0.4exp(-0.5|V|) (black line); d) inter-event interval I distribution (gray) with a fitted function ~ (exp(-0.2I)-exp(-0.3I)).

Irregular sampling is an obstacle for coupling analysis, where one typically presumes a time series sampled equidistantly in time. For this reason, we perform a preliminary smoothing of the data over intervals of about 5 years to match the temporal resolution of the used solar activity proxy. Moreover, this averaging interval may somewhat reduce the dating error effect. For smoothing we use a Gaussian kernel filter [S19] which appears somewhat better than the ordinary moving average, since it results in less fluctuating estimates as our experience shows. The data at the output of the Gaussian kernel filter read $x_k = \sum_n w(t_k, \tilde{t}_n)\tilde{x}_n$ where the weights are $w(t_k, \tilde{t}_n) = Ce^{-(t_k - \tilde{t}_n)^2/2\sigma^2}$, t_k is the time instant at the center of the averaging interval corresponding to x_k, σ is an efficient width of the averaging interval, C provides the normalization of the weights to unity. The value of $\sigma = 2.5$ years provides an approximately 5-yr weighted averaging. The result is shown with the red line in Fig. S1,a. The autocorrelation time of the 5-yr averaged signal is equal to 25 years if defined as the decay time of the autocorrelation function (ACF) fitted to small time lags (Fig. S1,b).

The TSI data are $y_n = y(t_n)$ where $t_n = t_0 + n\Delta t$ with $\Delta t = 5$ yrs and observation instants ranging from thousands years ago to 2007.5 AD. Each sample stands for a ΔTSI value averaged over a time interval (of more than 5 yrs width) centered at the corresponding time instant. By construction of the data, the 11-yr cycle is averaged out of them [S16]. The time series is plotted in Fig. S2,a over the period (50 BC – 2010 AD). The ACF for this solar proxy decays in about 30 yrs (Fig. S2,b). These data allow us to analyze the influence of relatively slow (20-30 yrs) solar activity variations on the similar scale climate variations found in the speleothem proxy record.

The volcanic activity data *V* are available as values of global atmospheric loading induced by all volcanic eruptions globally, including those in NH, in SH, and the tropics. Its time series looks like a sequence of pulses of different amplitude coming at random time instants (Fig. S3,a, black line). The distributions of pulse amplitudes and interpulse intervals along with their fits are presented in Fig. S3,c,d. The original index of volcanic activity $\tilde{y}_n = \tilde{y}(n\Delta t)$ is sampled at $\Delta t = 1$ yr. It equals *V* for the years of volcanic eruptions and zero otherwise. For the uniformity of the coupling analysis and possibility of simultaneous trivariate coupling analysis, we use $y(t)$ smoothed by Gaussian kernel with $\sigma = 2.5$ yrs, whose sample ACF is shown in Fig.S3,a. Dataset smoothing reduces the impacts of possible chronological uncertainties and minimizes potential noise associated with "coupling versus lag" tests. However, coupling estimates do not strongly depend on this smoothing, and are only weakly sensitive to the smoothing kernel width (provided it is less than about 5 yrs).

S3. Coupling between solar activity and $\delta^{18}O$ variations, asymptotic tests for significance

S3.1. CCF estimation. The CCF estimate over the entire interval of the last two millennia is shown in Fig.S4,b (black line). Its squared value (red line) is the coefficient of determination, i.e. the part of the variance explained by the linear regression of one signal onto the other. The maximum value of CCF is positive and reaches 0.3 at the time lag $l = 0$, so the coefficient of determination for the simple regression is only 10 %. The number of decorrelated segments within the time series (i.e. effective degrees of freedom in the *Z*-test) is $M \approx 2000/25 = 80$. Then, according to the Z-test, the point-wise (fixed-lag) significance level for rejecting the hypothesis of zero CCF at zero lag is $p = 0.012$. After the Bonferroni correction for multiple testing at various time lags (only the time lags separated by more than 25 yrs represent different tests), the final significance level for rejecting the hypothesis of zero CCF is $p = 0.05$. The Bonferroni correction is applied in the same way for the time-lagged WG causality below.

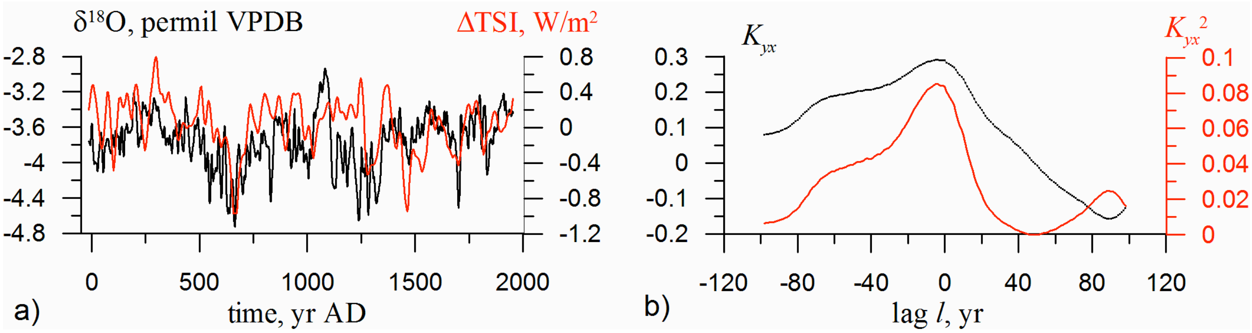

Figure S4. TSI variations [S16] and $\delta^{18}O$ variations [S14,S15,S18]: a) the data together; b) CCF (black) and squared CCF (red).

We note that it would be incorrect to interpret zero lag of the maximum CCF directly as zero delay time in the coupling mechanism, because the location of the CCF maximum is also affected by both characteristic time scales of the processes under study. In particular, if a first-order stochastic process y drives a first-order stochastic process x and both processes are characterized by an autocorrelation time τ, then the maximum of CCF should be achieved at a positive time lag of $\tau/2$. Thus, without dating errors we would expect the CCF maximum at a positive time lag of approximately 15 yrs. The dating errors can also shift the CCF maximum.

S3.2. Moving window CCF analysis. It is natural to suspect that CCF can change in time distorting the results of the above analysis which assumes stationarity of the processes under study. Therefore, a similar analysis in consecutive time windows (W–L, W) of some length L is relevant to account for possible non-stationarity. Such estimates are shown in Figure S5 (and Figure 5 in the main text). One can see larger values of CCF at time lags of –10 to –15 yrs for the earlier time windows (35 BC – 1035 AD) to (435 – 1435 AD). CCFs decrease for later windows. Note that the maximum CCF is achieved at positive time lag for the recent windows, while it is achieved at a negative time lag for earlier windows. The latter may well be due to larger dating errors for the earlier data.

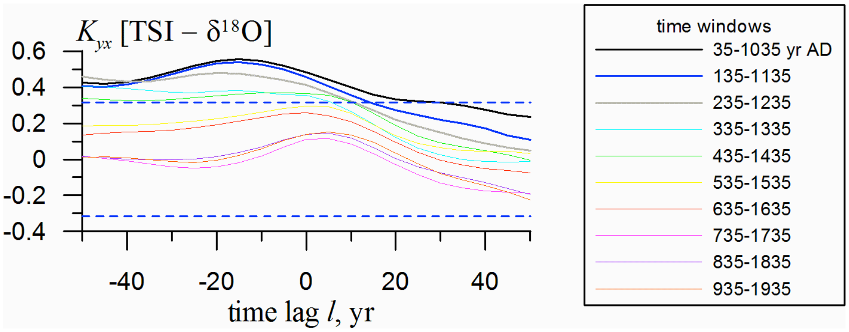

Figure S5. Moving window analysis of the CCF between ΔTSI and δ^{18}O variations (L = 1000 yrs), different colors are explained in the legend. The dashed lines show 95% critical values.

Significance levels and critical values for rejecting the null hypothesis of zero CCF within each separate window (fixed-window p-levels) are estimated via the Z-test as above. The critical value corresponding to the fixed-window p-level of 0.05 is shown with the dashed lines in Figure S5. After correction for multiple time windows (2000/1000=2 non-overlapping time windows), the CCF estimates are highly confident for the time windows up to (250 AD – 1250 AD) (Figure 5 in the main text). As for the maximal value of CCF about 0.6 achieved for the windows close to (50 AD – 1050 AD), after correction for multiple time lags and windows, it differs from zero at $p < 0.004$. Estimates for other window lengths (700 to 900 yrs) give similar results.

Thus, the presence of coupling between these two signals is established with high confidence based on the CCF estimates for the entire 2000-yrs period ($p < 0.05$). The moving window-based CCF estimates lead to a considerably higher confidence ($p < 0.004$) suggesting that the coupling between the processes may well change in time which makes the moving window analysis more sensitive. If so, the CCF analysis suggests that this coupling decreases over time from larger values over, roughly speaking, the first millennium AD to considerably smaller values over the second millennium AD.

As noted above, 10 % of the δ^{18}O time series variance is explained by a linear regression onto simultaneous TSI values (Fig. S4,b). If the residuals of such regression (which are obtained

via subtracting the respective regression values from the $\delta^{18}O$ time series) were delta-correlated, one could naturally assume that the $\delta^{18}O$ time series is just a superposition of the solar activity variations and some "rapidly fluctuating noise". However, we find that the time series of residuals is quite similar to the $\delta^{18}O$ time series and its ACF is close to the ACF of the $\delta^{18}O$ time series. Thus, in our case we have sufficient evidence to hypothesize that the $\delta^{18}O$ fluctuations result from the solar activity influence on another (regional climate) process with a characteristic time scale of about 25 yrs. Then, the Wiener-Granger causality is a relevant coupling characteristic to be used to improve the sensitivity of the tests as compared to the CCF analysis.

S3.3. Wiener-Granger causality estimates from the entire time series. The estimates of the WG causality $G_{y \to x}$ are based on fitting the bivariate autoregressive models of the form

$$x(t+\tau) = \sum_{k=1}^{d_x} a_k x(t-(k-1)\Delta t) + \sum_{k=1}^{d_{xy}} b_k y(t-(k-1)\Delta t-(s-1)\Delta t) + \xi_k(t), \qquad \text{(S1)}$$

and univariate autoregressive models of the form

$$x(t+\tau) = \sum_{k=1}^{d_x} a_k x(t-(k-1)\Delta t) + \xi'_k(t), \qquad \text{(S2)}$$

where τ is prediction time, s is relative temporal shift, ξ_k and ξ'_k are white noises whose variances are responsible for the residual prediction errors of both models. In order to assess statistical significance of the prediction improvement $G_{y \to x}$ at a given time shift s (i.e. to get a pointwise significance level p), we use the F-test which is based on the following fact: for $\tau = 1$ sampling interval (time step of the data used) and uncoupled processes x and y the statistic $\frac{N}{d_{yx}} \frac{\sigma_x^2 - \sigma_{x|y}^2}{\sigma_{x|y}^2}$ (where N is roughly the number of data points used to fit the AR-models, more precisely – the number of time instants for which the residual prediction errors are computed) is distributed according to the Fisher F-law with (N, d_{yx}) degrees of freedom. If the actual obtained value of this statistic exceeds (1-p)-percentile of that distribution then we reject the null hypothesis (infer the presence of coupling) at the significance level p.

For our data under study, N is roughly the number of 5-yr intervals covered by the time window used for the WG causality estimation (it is about 400 data points for the entire 2020-yr time series). The original YOK-I data are smoothed only over 5-yr intervals and the time series x used for the coupling estimation is sampled also at 5-yr intervals. Thus, no "excessive smoothing" seems to be introduced. Still, to further check this point we analyzed the results for greater prediction times as well. For $\tau > 1$, an effective number of non-overlapping prediction intervals for the AR-models is τ times smaller, so for uncoupled processes we assume the above statistic to be distributed according to the F-law with $(N/\tau, d_{yx})$ degrees of freedom. Thereby, we get a conservative estimate of the pointwise p-level. The results of significance level estimation are quite close for different values of τ (Figure S6) confirming that the time series of YOK-I is not excessively smoothed, i.e. no especially low p-level estimate are obtained for $\tau = 1$ as compared to the cases of greater τ.

Figure S6 shows the estimation results for the prediction times $\tau = \Delta t,\ 2\Delta t,\ 3\Delta t, 4\Delta t$ where the latter is close to the decorrelation times of the processes under study. At $\tau = \Delta t = 5$ yrs, the optimal (according to the Schwarz criterion) individual model order is $d_x = 4$. As for the dimension d_{xy}, its optimal value varies typically between 1 and 2 for different s and τ. The value $d_{xy} = 1$ leads to smoother plots of PIs and gives smaller significance level estimates. Therefore, it is always used below.

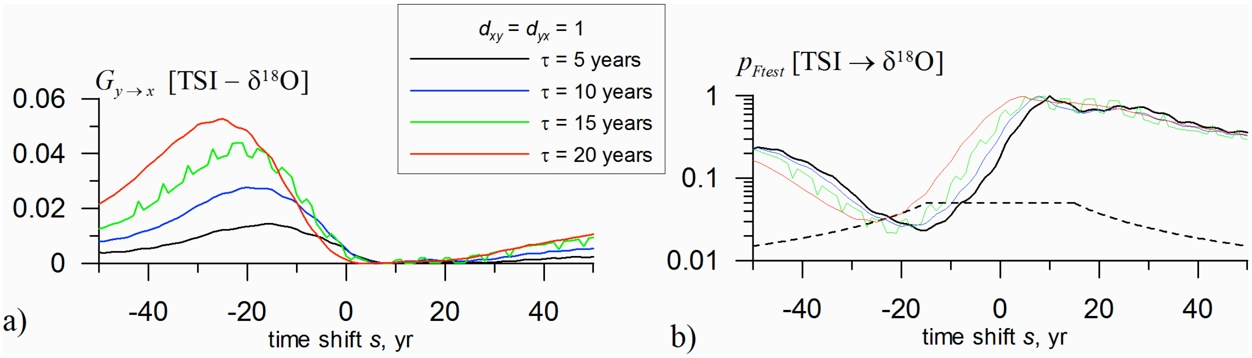

Figure S6. Wiener-Granger causality estimates for the pair "TSI – δ^{18}O" from the entire period for different prediction times τ (a) and the respective F-test-based point-wise significance levels (b) versus time shift. The dashed line in panel (b) show the point-wise significance level for $\tau = 5$ yrs which correspond to the significance level of 0.05 after the Bonferroni correction, assuming that the multiple correction is not needed for the time shifts around zero.

The plot for $G_{y\to x}$ at $\tau = 5$yrs (Figure S6,a, black line) exhibits maximum of $G_{y\to x} = 0.015$ at illogical time shift of –15 yrs indicating a dating error of this size (most probably, for earlier pieces of data). The above $G_{y\to x}$ is significant at the point-wise (fixed-shift s) significance level $p = 0.023$, while the Bonferroni correction for various s suggests to multiply this value by the number of independent s-intervals over the interval (–15, 15) yrs, i.e. by 30/25=1.2, that retains almost the same final $p < 0.03$. This illustrates an improved sensitivity of the test as compared to the above CCF estimate which differs from zero at $p < 0.05$. Larger prediction times do not improve the significance of the conclusions as compared to $\tau = 5$ yrs, since the variance of the estimators rises along with their values (not shown). Our numerical simulations show that $\tau = 5$ yrs give the best sensitivity of the tests, though the results for $\tau = 10$ yrs appear very similar. For brevity, we report only the results for $\tau = 5$ yrs below.

S3.4. Moving window Wiener-Granger causality analysis. Such an analysis is done for time windows of the lengths L = 1000, 900, 800, 700, and 500 yrs which are moved in steps of 25 yrs. In addition to Figure 4 in the main text, we can note similar results for the window lengths from 700 to 900 yrs with the most significant results achieved at L = 800 yrs. To summarize the results for the latter, $G_{y\to x,\max}$ are greatest for earlier windows and significant at the resulting significance level of $p < 0.05$ up to the window (300 AD – 1100 AD). Significant maximal PIs for earlier time windows correspond to the time shift of about s = –25 yrs up to the window (450 AD – 1250 AD). After that early period, $G_{y\to x,\max}$ is not significant. The maximal value of $G_{y\to x,\max} = 0.1$ achieved for the window (85 AD – 885 AD) is significant at the point-

wise (fixed-shift and fixed-window) p-level of 0.00005, and after the Bonferroni corrections at the resulting p-level of 0.0005. Other window lengths give similar results. The Bonferroni correction for the multiple testing over different L gives the resulting $p < 0.002$. This is by an order of magnitude less (i.e. more reliable) than the p-level for the entire window estimation.

The moving window estimates reveal a clear tendency of the TSI-to-δ^{18}O influence to decrease with time. The transition from strong to weak $G_{y\to x,\max}$ is sufficiently sharp: it starts from the window (300 AD – 1100 AD) and finishes for the window (500 AD – 1300 AD); a discussion of the most probable transition time (1100 – 1300 AD) is given in the main text. This agrees with the CCF analysis, but the transition between the two epochs revealed by the Wiener-Granger causality is sharper. It makes sense taking into account possible higher sensitivity of the Wiener-Granger causality to variations in coupling parameters. Testing the hypothesis of time-varying couplings with Monte Carlo simulations is presented below.

S4. Coupling between solar activity and δ^{18}O variations, Monte Carlo tests for significance

S4.1. CCF estimation. For uncoupled processes (1) (see "Data and methods" section in the main text), i.e. for $c = 0$, the estimate $\left|K_{yx}\right|_{\max}$ ranges from 0.04 to 0.45. The value of 0.3 obtained for the climate data rejects the hypothesis of uncoupled processes at $p = 0.07$. It agrees reasonably well with the Z-test p-level of 0.05. Figure 4b in the main text shows $\left|K_{yx}\right|_{\max}$ in moving windows of different lengths for the climate data (solid lines) and 0.95 percentiles of $\left|K_{yx}\right|_{\max}$ obtained from the model ensemble with the corresponding time series length (dashed lines). The window lengths in the range 700 to 1000-yrs reveal that the CCF for the paleoclimate data is significantly different from CCF estimates for the model at $p < 0.05$ for several early time windows. The 500-yrs window-based CCF estimates are not significantly different from the model CCF for uncoupled processes. Thus, the 500-yrs window-based estimates of CCF are not informative and 700 years is the minimal window length to be used. The best significance level is $p = 0.03$ for the window length of $L = 900$ and 1000 yrs. Accounting for multiple tests with window sizes, we get $p = 0.05$. All these numbers differ by an order of magnitude from the above Z-test which gives us $p < 0.007$. Hence, the Z-test may be too rough for the time series of the length of 1000 yrs and less, though it is sufficiently accurate for the time series length of 2000 yrs. Anyway, the CCF estimates finally allow us to detect coupling between the TSI and δ^{18}O variations at the resulting significance level of (at worst) $p = 0.05$.

Since the moving window analysis with $L = 700$ to 1000 yrs gives more significant results than that for the entire time series, we checked whether the model (1) with constant parameters (including the coupling coefficient c) agrees with the data properties. We tried different values of coupling coefficient $c\delta t$ (small integration step is $\delta t = 1$ month) and found that for the entire time series the model with $c\delta t = 0.0015$ gives CCF estimates whose median value is close to the empirical CCF value. According to the $\left|K_{yx}\right|_{\max}$ maximized over moving windows, the empirical CCF gets closer to the model (1) with $c\delta t = 0.002$ for $L = 1000$ yrs and even to the model with $c\delta t = 0.0025$ for $L = 700$ yrs. This suggests that certain time intervals may correspond to larger CCFs than the others. To check whether we can reject the hypothesis of the model (1) with constant parameters, we compute the CCF maximal difference (i.e. the range of $\left|K_{yx}\right|_{\max}$ values)

over all time windows W_k of the length L within the 2020-yrs interval, i.e. $\Delta\left|K_{yx}\right|_{\max} = \max_{W_k,\,|W_k|=L}\left|K_{yx}(W_k)\right|_{\max} - \min_{W_k,\,|W_k|=L}\left|K_{yx}(W_k)\right|_{\max}$. In particular, the hypothesis is rejected at the level of $p = 0.06$ for $L = 800$ yrs and for various coupling coefficients.

S4.2. Accounting for sampling irregularity and dating errors. To assess the role of dating errors, we perform the same tests with the model (1) where the dates of all irregularly sampled X-values were noise-corrupted. Namely, we assume that the dating errors are small for the recent data but rise with the age, in accordance with their estimated errors. It seems that the Brownian motion model (Wiener process) is appropriate to capture these characteristics. Namely, as a basic case, we assume that the dating error variance rises at the rate of 0.025 yr^2/yr, starting from zero at zero age. Then, for the age of 2000 yrs one gets the variance of 50 yr^2, i.e. the standard deviation of the dating error of 7 years and 95% confidence interval of the width ± 14 yrs which agrees reasonably well with the original estimates [S14].

It appears that the influence of such dating errors on the above results is negligible. First, the CCF estimates for zero coupling in the model (1) are indistinguishable for the cases of dating errors and precisely dated X-values, as can be expected since ACF properties of the data are not affected by such moderate dating errors. One may expect that dating errors should reduce the values of CCF in case of non-zero coupling. However, the above level of dating errors reduces the mean value of CCF estimates for the model (1) with $c\delta t = 0.0015$ (over an ensemble of 1000 time series) only by 1 %. Such dating errors also do not change the above significance levels of the nonzero CCF detection and the CCF temporal variation detection. We have also checked stronger dating errors: twice and four times as big dating error standard deviation, i.e. the dating error variance rising with age at the rate of 0.1 and 0.4 yr^2/yr. For 2000-yrs ages, it gives the dating error standard deviations of 14 and 28 years, respectively. These large values do not change the CCF estimates for uncoupled processes and, hence, do not affect the test against zero CCF. As for the coupled processes with $c\delta t = 0.0015$, the 14-yr dating error reduces the mean value of CCF estimates approximately by 5 % and 28-yr dating error by about 10 %. However, the significance levels of the conclusions are not affected (within statistical fluctuations).

The weak influence of the dating errors can be understood from the following considerations. First, these errors lead mainly to the shift of large portions of the time series, leading mainly to a change in the time lag corresponding to the maximum CCF (± 14 yrs, i.e. well within 20 yrs for the earliest dating error level, consistent with the data at hand). However, we maximize over time lags and, hence, such a shift does not influence the result. Next, the autocorrelation time of the processes is about 20 to 25 yrs and, further, a 5-yrs kernel filtering is performed, giving the autocorrelation times of the resulting signals of about 25 to 30 yrs. Thus, CCF for the time lags separated by less than 20 years are close to each other. Hence, the CCF values and the plots of CCF versus time lag can be only weakly affected by the dating errors which are less than 20 yrs. Some effects (about 5-10% even for much larger dating errors) can be seen, but they are masked by statistical fluctuations determined by the shortness of the time series (about 70-100 characteristic times). Therefore, the resulting significance levels of the tested hypotheses do not depend on the dating errors in simulations of the model (1). Hence, the YOK-I $\delta^{18}O$ variations are quite precisely dated and one could even ignore dating errors in the significance tests with the model (1).

Thus, we have established the coupling presence between TSI and $\delta^{18}O$ variations at least at the level of $p = 0.05$, using both asymptotic and Monte Carlo-based significance tests with CCF. We have also rejected the hypothesis of a constant CCF (constant model parameters) with reasonably high confidence (at $p < 0.07$, Monte Carlo-based test). We conjecture that the coupling (in terms of CCF values) between the solar activity and $\delta^{18}O$ variations was maximal over the period (100 AD – 900 AD) and decreased with time from larger values over the first millennium AD to smaller values over the second millennium AD.

S4.3. Wiener-Granger causality estimation. Let us now compare the Wiener-Granger causality estimates from model (1) time series to the respective estimates obtained from the paleoclimate data and check whether we can reject the hypothesis (i) of uncoupled processes and (ii) of the processes with constant coupling. We fix $d_x = 4$ and $d_{xy} = 1$, estimate $G_{y \to x,\max}(W_k)$ for a concrete time window W_k of the length L and maximize over all subsequent windows within the 2020-yr interval to get $G^{\max}_{y \to x} = \max\limits_{W_k, |W_k| = W} G_{y \to x,\max}(W_k)$. We also compute the difference between maximal and minimal values of $G_{y \to x,\max}(W_k)$ to estimate the range $\Delta G_{y \to x,\max} = \max\limits_{W_k, |W_k| = W} G_{y \to x,\max}(W_k) - \min\limits_{W_k, |W_k| = W} G_{y \to x,\max}(W_k)$. All tests are performed for L = 2020, 1000, 900, 800, 700, and 500 years.

For the real-world data above we have obtained $G^{\max}_{y \to x} = 0.014$ at L = 2020 yrs. Analyzing a 1000-member ensemble of realizations of the model (1) with zero coupling, we find that the above value of $G^{\max}_{y \to x}$ differs from zero only at p = 0.2 (i.e. non-significantly). The moving window analysis gives more informative results as shown in Figure 4a (main text). In addition, we computed the probability for the model $G^{\max}_{y \to x}$ estimates (at different coupling coefficients) to exceed the climate data $G^{\max}_{y \to x}$ estimates from 1000-member ensemble of realizations. For the uncoupled model (1) this is a p-level at which the *no coupling* hypothesis is rejected. After the Bonferroni correction, it equals p = 0.03. This is an evidence in favor of non-constant coupling hypothesis and relevance of shorter (700 to 1000 yrs) moving windows than the entire 2020-yrs interval, similarly to the consideration in Section S4.1.

The results of the tests against the model (1) with constant parameters (including the above "best-fit" coupling coefficient $c\delta t = 0.0015$) on the basis of $\Delta G_{y \to x,\max}$ are described in the main text. Namely, we rejected the hypothesis of constant parameters and, hence, inferred a significant decrease in the coupling characteristics over the interval under study at p = 0.1 for a window length of L = 800 yrs. This is somewhat less significant than p = 0.06 for the CCF change detection. Thus, the Wiener-Granger causality analysis is more confident in respect of the coupling presence and the "transition point" in the coupling strength than the CCF analysis. However, the latter more confidently detects the presence of temporal variations in coupling.

To summarize, for the pair ΔTSI-$\delta^{18}O$ the time windows of 700-1000 yrs length allow us to infer coupling presence at the level of p = 0.03-0.05 (coupling estimates are especially large for the windows (100 AD – 900 AD) to (250 AD – 1050 AD) based on the WG causality estimates) and to detect the coupling decrease at p = 0.06-0.1. Most probably, the change in the parameters

of the processes determining the change in the coupling characteristics occurred between 1000 AD and 1300 AD (Figure 4a in the main text).

S5. Testing robustness of the results with different isotopes and solar proxies

Coupling between TSI and $\delta^{13}C$ variations (Figure 5a, main text) was analyzed analogously to the TSI-$\delta^{18}O$ pair. The results were overall similar with less confident conclusion for the TSI-$\delta^{13}C$ pair (Figure 5b). Both the similarity and the difference are not surprising. Indeed, there is considerable correlation between the two isotope records: their CCF achieves a maximum of 0.7 at zero time lag. Despite the cross-correlation is large, it corresponds to the situation when only half the variance of one signal is explained by regressing it on the other. Thus, significant differences between the two isotope records are also present so one could expect somewhat different results of the coupling analysis as well. The presence of the TSI-$\delta^{13}C$ coupling is detected best for the window length L = 1000 yrs at p = 0.006 according to the Bonferroni corrected F-test. However, Monte Carlo tests with the uncoupled model (1) give at best p = 0.2. Still, an overall character of the temporal evolution of $G_{y\to x,\max}$ (Figure 5b, red line) is similar to that for the TSI-$\delta^{18}O$ couplings (Figure 5b, blue line).

The coupling between another solar activity proxy (-$\Delta^{14}C$ variations compiled from different natural archives, IntCal13 calibration curve [S20], Figure S7, black line) and both YOK-I isotope records was performed along in the same way as above. The difficulty here lies in the fact that the IntCal data exhibit stronger slow components with time scales about 500 yrs (e.g. the decrease of the solar activity proxy since 1250 till 1750 AD) which cannot be easily removed. We performed the analysis of the original data including such trends, so the slow components may well influence the results and their statistical significance. The presence of coupling between this solar activity proxy and $\delta^{13}C$ variations is detected best for window length L = 800 years at p = 0.0015 according to the Bonferroni corrected F-test. Monte Carlo tests with the uncoupled model (1) with τ_x = 25 yr and τ_y = 172 yr (accounting for longer autocorrelations) give p = 0.043 which is still significant. Moreover, the maximum of $G_{y\to x}(s)$ is achieved at positive (i.e. physically meaningful) time shift of s = 10 years, at variance with the negative shift for the above TSI reconstruction. This finding further justifies our conclusion about accurate dating of the YOK-I data and possible dating error in the TSI reconstruction.

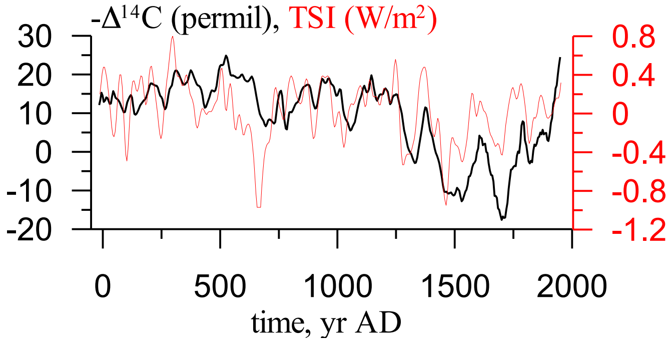

Figure S7. Solar activity proxies: TSI reconstruction (red line) and IntCal13 data (black line).

An overall character of $G_{y\to x,\max}$ temporal evolution (Figure S8, black line) is similar to that for the above results for the TSI-$\delta^{13}O$ pair (Figure S8, red line). Monte-Carlo tests with

the model (1) reject the hypothesis of constant parameters at $p < 0.06$ for $L = 800$ yr and less confidently at other window lengths. So we can infer the coupling decrease only at $p = 0.1$ after the Bonferroni correction for different window lengths. Still, this result is close to what is obtained for the TSI data, even though somewhat less confident. However, it is important to note that the maximal WG causality for early windows is achieved at physically meaningful positive lag of 10 yr (Figure S9). This stays in contrast with the negative lag for the TSI reconstruction (Figure 2a in the main text). Hence, this result suggests accurate dating of the YOK-I and Intcal13 data over the first millennium AD supporting the conclusion about the dating error in the TSI reconstruction over the first millennium AD.

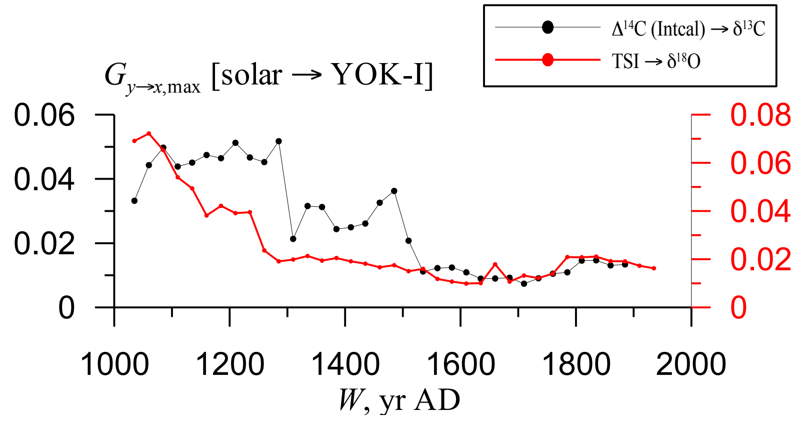

Figure S8. Moving window Wiener-Granger causality estimates between IntCal13 solar activity proxy and $\delta^{13}C$ variations (black line) versus the window endpoint. For convenience they are compared to the results for the TSI-$\delta^{18}O$ pair (red line).

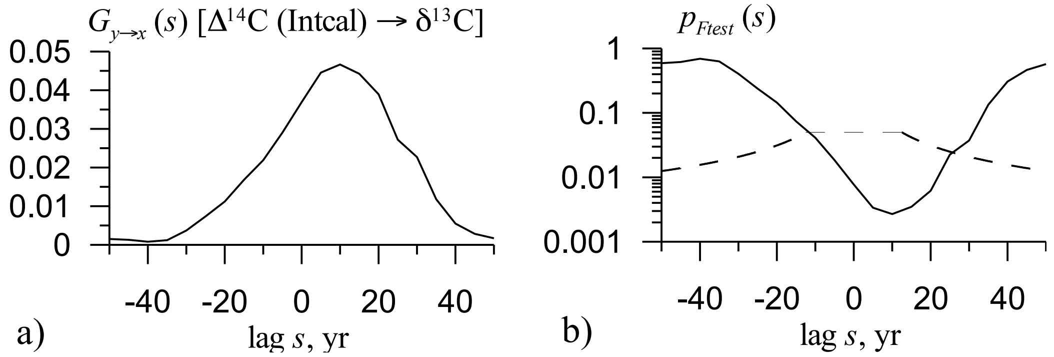

Figure S9. Wiener-Granger causality estimates for the Intcal13 solar activity proxy and $\delta^{13}C$ variations in the time window (235 – 1235 AD) versus temporal shift s between the time series (a) and the F-test significance level (b). Positive s corresponds to moving $\delta^{13}C$ data to the past.

As for the coupling between the Intcal13 data and $\delta^{18}O$, its estimates are hardly significant. Only weak signs of the coupling decrease from 1100 CE to 1300 AD are seen (Figure S10, red line). Thus, the overall conclusion about coupling between the Intcal13 data and YOK-I data appears less confident than that for the TSI reconstruction, though their qualitative agreement is clearly observed. Moreover, physically meaningful positive time shift corresponds to the maximal WG causality characteristic. In total, these additional data analysis supports the conclusions presented in the main text based on the data for the TSI and $\delta^{18}O$ variations.

To compare the above results to other stalagmite records from Central America or Tropical Africa and other solar reconstructions seems currently impossible, because such data for our analysis must satisfy the following requirements: (i) cover at least the last two millennia; (ii) possess temporal resolution not worse than 1 sample per 5 years; (iii) be free from strong slow components (with characteristic time scales of 500 years and more). To our best knowledge all records currently available fail to satisfy at least one of these requirements, e.g. the stalagmite data from the Tamboril Cave (Brazil) [S21] have sufficient temporal resolution only over the last millennium while their resolution for the ages of 1000-1700 yrs BP ranges from 10 to 15 yrs which is not enough for a clear comparison; the stalagmite data from the Huagapo Cave (Peru) [S22] exhibit strong trends over time scales from 200 yrs to 700 yrs and, moreover, consist of two pieces which overlap over the ages of 1100-1400 yrs BP and do not coincide with each other so it is not clear how to remove the trends and convert the data into a single piece. Many of the other stalagmite records available are just not long enough. Very similar problems, especially concerning temporal resolution, are encountered for other available solar reconstructions.

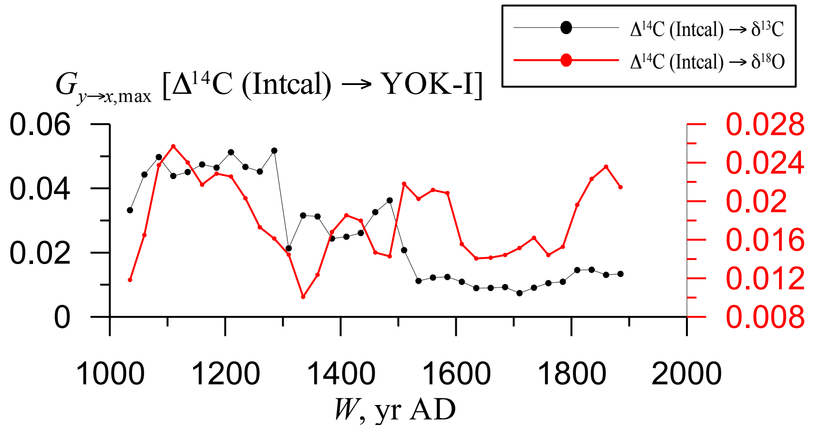

Figure S10. Moving window Wiener-Granger causality estimates between IntCal13 solar activity proxy (black line) and $\delta^{13}C$ variations in comparison to the Intcal13-$\delta^{18}O$ pair (red line).

S6. Estimation of coupling between volcanic activity and δ^{18}O variations

We analyzed the coupling between volcanic activity (Figure S3,a) and $\delta^{18}O$ variations according to the same procedure as described above for the study of the TSI influence. The most significant results are obtained for time windows of the length L = 1000 yrs. The CCF estimates (Figure S11a) are significantly different from zero, e.g. the maximal CCF about 0.3 is achieved for the window (735 AD – 1735 AD) and allows us to reject the no coupling hypothesis at the Bonferroni corrected Z-test based significance level $p < 0.004$. The Wiener-Granger causality characteristics appear even more sensitive (Fig. S11b,c). This can be expected, since the volcanic activity variations $y(t)$ are faster than the 25-yr time scale of the x-signal. Thus, quite a strong influence would be necessary to provide considerable cross-correlations. At the same time, a moderately strong influence can be readily seen from the fitted AR-models with the prediction time τ = 5 yrs. The WG causality estimates are for almost all windows are quite significant. Namely, $G_{y\rightarrow x,\max} \approx 0.1$ is achieved for the window (235 AD – 1235 AD) and significant at the Bonferroni corrected F-test based level of $p < 0.0004$. Moreover, the value of $G_{y\rightarrow x}(s)$ reaches its maximum at (physically reasonable) positive time shift s of 2-3 yrs confirming accurate

dating of both data sets. The window lengths of L = 800 yr and L = 1200 yr give very similar results with slightly less significant rejections of the *no coupling* hypothesis (not shown).

Monte-Carlo tests for significance with L = 1000 yrs against the hypothesis of uncoupled processes (2) gave the following results. The dashed line in Figure S12b shows the point-wise p-level which corresponds to the rejection of the *no coupling* hypothesis at the final $p < 0.05$. It appears that the paleoclimate data estimate $G_{y \to x,\max} \approx 0.1$ allows us to reject the hypothesis of uncoupled processes (2) at the level of $p < 0.001$ close to the above F-test. The hypothesis of constant parameters is not rejected by the Monte Carlo tests with the model (2) and constant nonzero c at a reasonably small significance level (like 0.1 or smaller) suggesting that the volcanic influence on climate remains more constant in time than the detected time-changing solar influence.

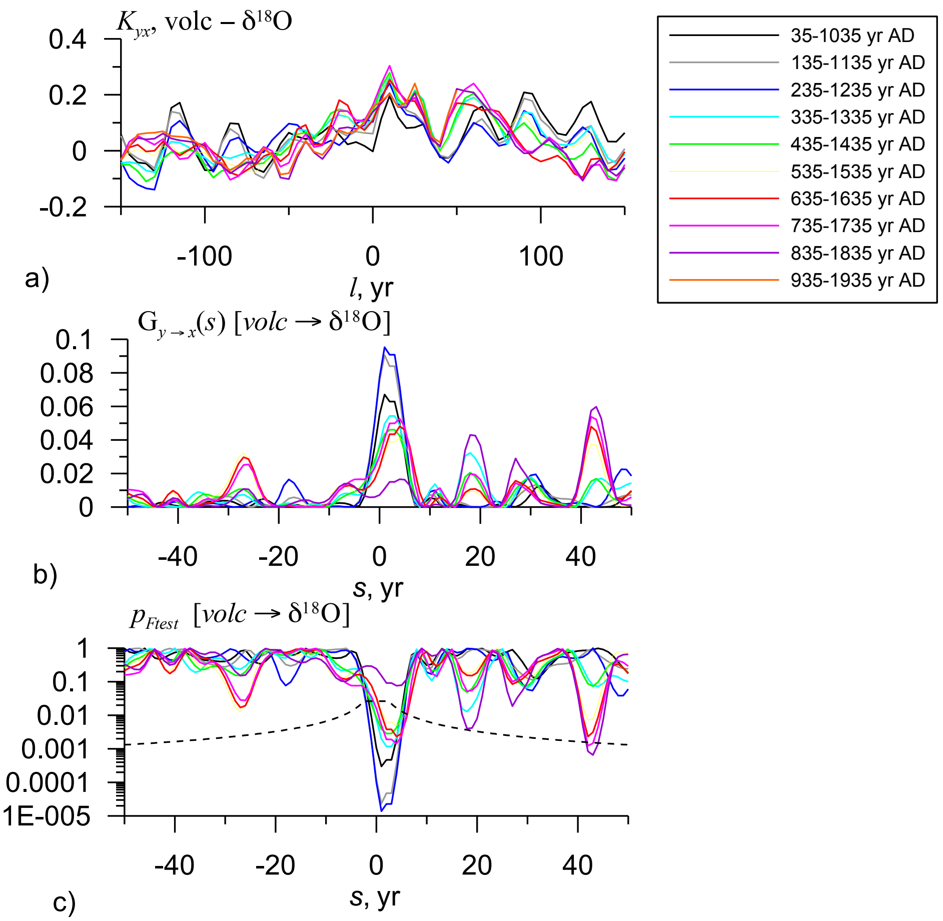

Figure S11. Coupling estimates between volcanic activity and local climate proxy: a) CCF; b) WG causality; c) F-test based p-level to reject the *no coupling* hypothesis, the dashed line shows the fixed-shift and fixed-window p-level which corresponds to the Bonferroni corrected $p = 0.05$.

Finally, we note that in addition to the above bivariate Wiener-Granger causality analysis, we have preformed an analogous trivariate analysis for the three processes at hand (solar and volcanic activity and climate variations). However, its results are almost indistinguishable from those presented above: the estimates of solar (volcanic) influence on climate under the condition

that the volcanic (solar) influence is taken into account are the same as the respective bivariate estimates. This can be intuitively understood, since the solar and volcanic activity records are not mutually correlated over the considered time intervals.

S7. Concluding remarks

We have detected influences of solar and volcanic activity variations on the climate variability (represented by the speleothem proxy from Yok Balum cave, Belize) using the Wiener-Granger and CCF analysis.

We have used both asymptotic and Monte Carlo tests to reject the hypotheses of "no coupling" and "constant coupling".

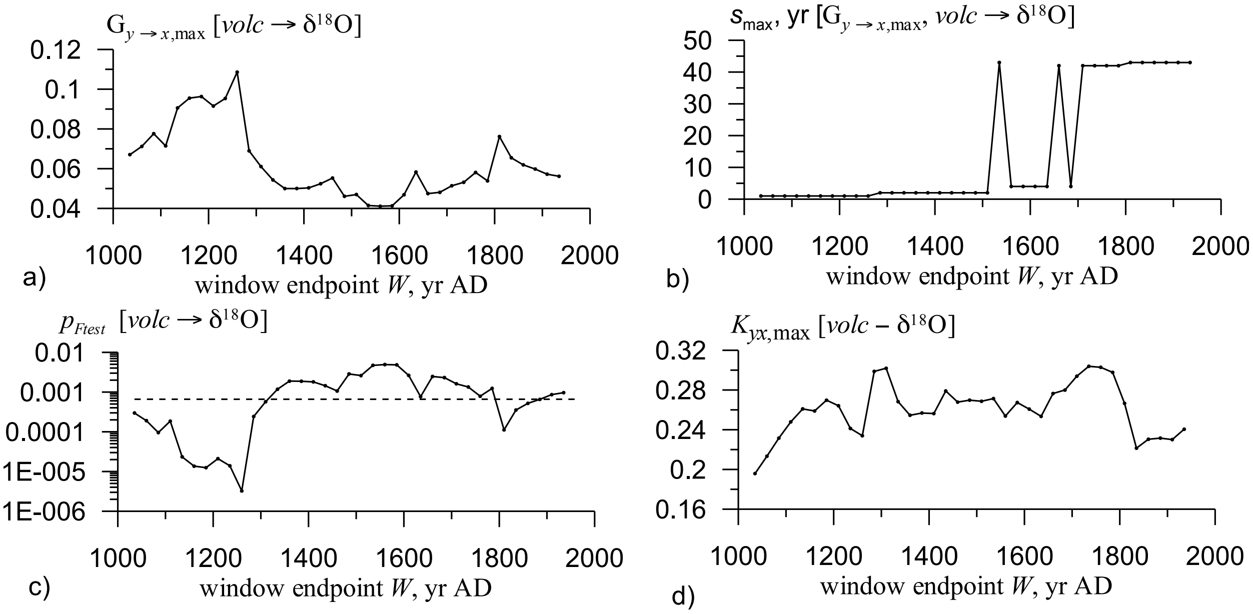

Figure S12. Moving window based couplings estimates between volcanic activity variations and local climate proxy: a) WG causalities, the dashed line show 0.95-percentile for the uncoupled model (2); b) time shift corresponding to the maximal WG causality; c) F-test based estimate of the pointwise significance level to rejecting the no coupling hypothesis, the dashed line shows the fixed-shift and fixed-window p-level which corresponds to the Bonferroni corrected $p = 0.05$.

Based on the moving window analysis of the Wiener-Granger causality, we infer a change (a considerable decrease) in the strength of the solar influence on climate in the second millennium AD in comparison with the first millennium AD. More concretely, we argue that the transition interval lies within 1000 – 1300 AD. Such a localization of the transition interval is possible only with the Wiener-Granger analysis, while the CCF estimation gives less definite results. This conclusion is confirmed by using either of the two isotopes records ($\delta^{18}O$ and $\delta^{13}C$) and either of the two solar activity proxies (based on ^{10}Be from ice cores and compiled from various sources).

Volcanic influence on climate seems to be more constant in time. With decreasing solar impact on regional climate, volcanic forcing increases relatively with the onset of the Little Ice Age (ca. 1300 AD), in line with earlier findings [S23].

One of the solar activity reconstructions (Intcal13 data [S20]) and volcanic activity data exhibit physically meaningful positive lags at which the WG causality from them to the YOK-I signals is maximal. Therefore, we infer sufficiently accurate dating of all these data. Since

another solar activity reconstruction [S16] exhibits illogical negative shift in the WG causality during the first millennium AD (and otherwise the most statistically significant results), we suggest that it may be corrupted with dating error of about 15 years for that early period. This conclusion is supported by further numerical analysis of the dating error problem in Ref. [S24].

References

[S1] Tol R. S. J. and A.F. De Vos (1993) Greenhouse Statistics – Time Series Analysis, *Theor. Appl. Climatol. 48*, 63–74.

[S2] Tol R. S. J. and A.F. De Vos (1998) A Bayesian Statistical Analysis of the Enhanced Greenhouse Effect. *Clim. Change, 38***,** 87–112.

[S3] Verdes P. F. (2005) Assessing Causality from Multivariate Time Series. *Phys. Rev. E*, *72*, 026222.

[S4] Verdes P. F. (2007) Global Warming Is Driven by Anthropogenic Emissions: A Time Series Analysis Approach. *Phys. Rev. Lett.*, *99*, 048501.

[S5] Lean J. L., and D. H. Rind (2008) How Natural and Anthropogenic Influences Alter Global and Regional Surface Temperatures: 1889 to 2006. *Geophys. Res. Lett.*, *35*, L18701. doi:10.1029/2008GL034864.

[S6] Mokhov I. I. and D. A. Smirnov (2008) Diagnostics of a Cause–Effect Relation between Solar Activity and the Earth's Global Surface Temperature. *Izvestiya, Atmospheric and Oceanic Physics*, *44*(3), 263–272. doi:10.1134/S0001433808030018.

[S7] Smirnov D. A. and I.I. Mokhov (2009) From Granger causality to long-term causality: Application to climatic data. *Phys. Rev. E*, *80*, 016208.

[S8] Mokhov I. I. and D. A. Smirnov (2009) Empirical Estimates of the Influence of Natural and Anthropogenic Factors on the Global Surface Temperature. *Doklady Earth Sci.*, *427*(1), 798–803.

[S9] Mokhov I. I., D. A. Smirnov, and A. A. Karpenko (2012) Assessments of the Correlation between Variations in the Global Surface Air Temperature and Different Natural and Anthropogenic Factors Based on the Data of Observations. *Doklady Earth Sci.*, *443*(1), 381–387.

[S10] Attanasio A. (2012) Testing for Linear Granger Causality from Natural/Anthropogenic Forcings to Global Temperature Anomalies. *Theor. Appl. Climatol.*, *110*(1-2), 281-289. doi:10.1007/s00704-012-0634-x.

[S11] Imbers J., A. Lopez, C. Huntingford, and M. R. Allen (2013) Testing the Robustness of the Anthropogenic Climate Change Detection Statements Using Different Empirical Models. *J. Geophys. Res. Atmos.*, *118*, 3192–3199, doi:10.1002/jgrd.50296.

[S12] Stern D. I. and R. K. Kaufmann (2014) Anthropogenic and Natural Causes of Climate Change. *Clim. Change*, *122*, 257–269, doi:10.1007/s10584-013-1007-x

[S13] Stips A., D. Macias, C. Coughlan, E. Garcia-Gorriz, and X. San Liang (2016) On the causal structure between CO_2 and global temperature**.** *Scientific Reports***,** *6*, 21691, doi: 10.1038/srep21691.

[S14] Kennett D. J., et al (2012) Development and Disintegration of Maya Political Systems in Response to Climate Change. *Science*, *338*(6108), 788–791, doi:10.1126/science.1226299.

[S15] Ridley, H. E. et al. (2015) Aerosol forcing of the position of the intertropical convergence zone since ad 1550, *Nat. Geosci.*, 8, 195–200, doi: 10.1038/ngeo2353.

[S16] Steinhilber F., J. Beer, and C. Fröhlich (2009) Total solar irradiance during the Holocene, *Geophys. Res. Lett.*, *36*, L19704, doi:10.1029/2009GL040142.

[S17] Sigl M. et al. (2015) Timing and Climate Forcing of Volcanic Eruptions for the Past 2,500 Years, *Nature*, *523*, 543–549, doi:10.1038/nature14565.

[S18] Kennett, D. J., S. F. M. Breitenbach, F. Lechleitner, H. E. Ridley, Y. Asmerom, V. Polyak, D. A. Smirnov (2017) Yok Balum Cave, Belize 2000 Year Stalagmite Stable Isotope Data. V. 2. GFZ Data Services. http://doi.org/10.5880/pik.2017.004.

[S19] Rehfeld K., N. Marwan, S.F.M. Breitenbach, and J. Kurths (2013) Late Holocene Asian summer monsoon dynamics from small but complex networks of paleoclimate data. *Clim. Dyn., 41*, 3–19, doi: 10.1007/s00382-012-1448-3..

[S20] Reimer P. J., E. Bard, A. Bayliss, J. W. Beck, …, J. van der Plicht (2013) Intcal 13 and Marine 13 Radiocarbon Age Calibration Curves 0–50,000 Years Cal BP, *Radiocarbon*, 55(4), 1869–1887; 10.2458/azu_js_rc.55.16947.

[S21] Wortham B. E., C. I. Wong, L. C. R. Silva, D. McGee, …, R. V. Santos (2017) Assessing response of local moisture conditions in central Brazil to variability in regional monsoon intensity using speleothem 87Sr/86Sr values, *Earth and Planetary Science Letters*, 463, 310-322; 10.1016/j.epsl.2017.01.034.

[S22] Kanner L. C., S. J. Burns, H. Cheng, R. L. Edwards, M. Vuille (2013) High-resolution variability of the South American summer monsoon over the last seven millennia: insights from a speleothem record from the central Peruvian Andes, Quaternary Science Reviews, 75, 1-10; 10.1016/j.quascirev.2013.05.008.

[S23] Miller G. H. et al. (2012) Abrupt onset of the Little Ice Age triggered by volcanism and sustained by sea-ice/ocean feedbacks, *Geophys. Res. Lett.*, 39(2), doi:10.1029/2011GL050168.

[S24] Smirnov D. A., N. Marwan, S. F. M. Breitenbach, F. Lechleitner, J. Kurths (2017) Coping with dating errors in causality estimation, *Europhys. Lett.*, 117, 100004; 10.1209/0295-5075/117/10004.
